# Effects of Yoga on Gene Expression: A Systematic Review of Randomised Controlled Trials

**DOI:** 10.7759/cureus.82690

**Published:** 2025-04-21

**Authors:** Selvaraj Giridharan, Soni Soumian, Nagaraj V Kumar, Jawaher Ansari

**Affiliations:** 1 Department of Oncology, Tawam Hospital, Al Ain, ARE; 2 Department of General Surgery, Tawam Hospital, Al Ain, ARE; 3 Department of Emergency, Tawam Hospital, Al Ain, ARE

**Keywords:** epigenetics, gene expression, inflammation, telomeres, yoga

## Abstract

Yoga, an integrative mind-body practice, is increasingly recognised for its ability to modulate gene expression, particularly that of genes associated with inflammation, stress, and aging. This systematic review, conducted per the Preferred Reporting Items for Systematic Reviews and Meta-Analyses (PRISMA) guidelines, synthesises evidence from 11 randomised controlled trials (RCTs) from 2015 to 2024, involving over 700 adults. Studies were sourced from PubMed, Scopus, Web of Science, and the Cochrane Library to evaluate the molecular effects and clinical implications of yoga. Across these RCTs, yoga consistently downregulated pro-inflammatory genes - interleukin-6 (IL-6), tumour necrosis factor-alpha (TNF-α), and nuclear factor kappa B (NF-κB) - in five studies, and upregulated anti-inflammatory and immune-regulatory genes - transforming growth factor-beta (TGF-β), forkhead box P3 (FoxP3), soluble human leukocyte antigen G (sHLA-G), and IL-10 - in four studies. It also enhanced the expression of genes linked to deoxyribonucleic acid (DNA) repair (OGG1, or 8-oxoguanine DNA glycosylase), mitochondrial function (adenosine monophosphate-activated protein kinase (AMPK), and sirtuin 1 (SIRT-1)), and epigenetic regulation (e.g., reduced TNF methylation and increased microRNA-133B (miR-133B)). These molecular changes were associated with clinical benefits, including reduced disease activity in rheumatoid arthritis (RA), improved glycemic control in type 2 diabetes (T2D), and improved quality of life among breast cancer survivors. Despite these promising findings, the small sample sizes and short intervention durations limit statistical power and generalisability. Yoga shows potential as a complementary therapy for managing inflammation and age-related conditions, but larger, longer-term RCTs with standardised protocols are essential to substantiate its therapeutic value and elucidate its mechanisms.

## Introduction and background

Yoga, an ancient practice with origins in India, has evolved from its spiritual beginnings to become a widely embraced phenomenon practiced by millions worldwide. Derived from the Sanskrit word "Yuj," meaning union or integration, yoga combines physical postures (asanas), breath control (pranayama), meditation (dhyana), and ethical principles to foster harmony between the body, mind, and spirit [1,2]. Historically developed as a path to enlightenment within traditions such as Hatha and Raja yoga, it has evolved across centuries and cultures, gaining widespread popularity in the West as both a fitness regimen and a therapeutic tool [3-6]. Yoga's holistic benefits - enhancing physical vitality, improving posture, regulating sleep, and reducing stress and anxiety [7-9] - underscore its enduring appeal. These advantages make yoga a promising complementary approach for managing conditions such as cancer, chronic pain, and cardiovascular disease [10-13].

Beyond these well-documented effects, recent scientific interest has turned to yoga’s influence at the molecular level, particularly its potential to modulate gene expression and epigenetic processes [14-16]. Advances in genomics have revealed that yoga may leave a "molecular signature," altering gene activity in ways that counteract the detrimental effects of chronic stress, a known driver of inflammation, aging, and disease [17,18]. Research highlights the ability of yoga to downregulate pro-inflammatory genes, such as nuclear factor kappa B (NF-κB), while upregulating genes linked to antiviral immunity, deoxyribonucleic acid (DNA) repair, and neuroprotection [19-21]. Additionally, yoga has been associated with increased telomerase activity, an enzyme critical for maintaining telomere length and promoting cellular longevity [22,23]. These molecular shifts are often accompanied by epigenetic changes, such as DNA methylation and histone modifications, which enhance the body’s resilience to stress-related conditions.

Despite a growing body of evidence, the precise mechanisms underlying the molecular benefits of yoga remain unclear. An earlier systematic review by Buric et al. synthesised pre-2015 studies on mind-body interventions, including yoga, and demonstrated the downregulation of inflammatory pathways [24]. However, recent advances in transcriptomics have enabled more precise gene expression analyses, necessitating a focused review of new randomised controlled trials (RCTs). Short-term studies provide promising insights, but uncertainties persist regarding the long-term effects of yoga on gene regulation, its efficacy compared with other interventions, and its applicability across diverse populations. These gaps underscore the need for a comprehensive synthesis to elucidate the therapeutic potential of yoga and guide its integration into modern healthcare.

This systematic review addresses this need by synthesising evidence from RCTs conducted between 2015 and 2024 to examine the effects of yoga on gene expression. By critically analysing the molecular pathways through which yoga confers health benefits, this study bridges ancient practices with contemporary science, clarifying yoga’s role as a complementary therapy. This review aims to inform clinical applications and set the stage for future research, establishing yoga as a scientifically validated approach for managing stress- and inflammation-related disorders.

## Review

Methods

This systematic review was conducted in accordance with the Preferred Reporting Items for Systematic Reviews and Meta-Analyses (PRISMA) guidelines to ensure transparency and reproducibility [25].

Eligibility Criteria

Studies were eligible if they were RCTs published between 2015 and 2024, involving human participants (healthy or with chronic diseases such as cancer, cardiovascular, or autoimmune disorders). Interventions focused on yoga or related mind-body practices (e.g., meditation and pranayama), excluding studies combining yoga with drugs unless the effects of yoga were isolated. Comparisons included standard care, no intervention, and active controls (e.g., exercise and health education). The primary outcomes were gene expression and epigenetic changes, with secondary outcomes including inflammation, stress, and longevity markers. Only English-language publications were included in this study. Observational studies, reviews, case reports, and non-RCTs were also excluded. Observational studies were excluded to prioritise causal evidence from RCTs.

Data Sources and Search Strategy

PubMed, Scopus, Web of Science, and the Cochrane Library were searched using the keywords (“yoga” OR “asana” OR “pranayama” OR “meditation”) AND (“gene expression” OR “epigenetics” OR “transcriptomics”), filtered for 2015-2024.

Data Extraction

Data were extracted using a standardised form, capturing the study design, participant characteristics (sample size, age, sex, and health status), intervention details (yoga type, frequency, and duration), and comparators. Primary outcomes focused on gene expression and epigenetics, while secondary outcomes included clinical and physiological changes. Two reviewers independently extracted data and resolved discrepancies by consensus. The consensus involved discussions to ensure accuracy. 

Quality Assessment

The Cochrane Risk of Bias tool evaluated randomisation [26], blinding, sample size, and confounders, with two reviewers independently assessing and resolving disagreements. No statistical tests (for example, Egger’s test) were used because of narrative synthesis, but potential bias was qualitatively assessed.

Synthesis of Results

Due to the heterogeneity in yoga styles, populations, and outcomes, a narrative synthesis summarised gene expression changes, clinical correlations, and study consistency. Heterogeneity was evaluated by comparing intervention types and molecular targets.

Results

**Figure 1:**
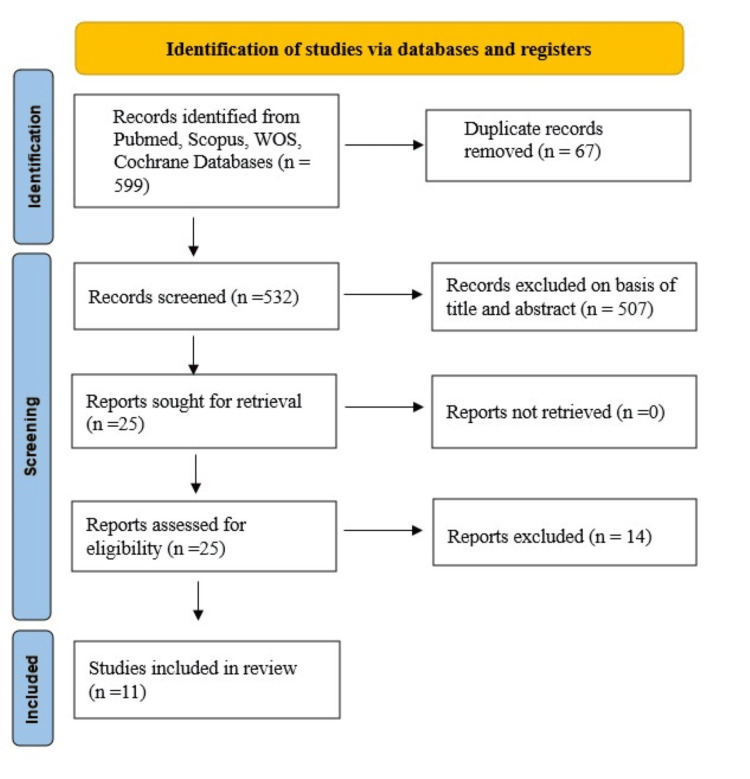
PRISMA flow chart for summarised search strategy PRISMA, Preferred Reporting Items for Systematic Reviews and Meta-Analyses

The search identified 599 records (300 from PubMed, 200 from Scopus, 80 from Web of Science, and 19 from Cochrane), with 67 duplicates removed and 532 screened by title/abstract. After excluding 507 records, 25 full-text articles were assessed, yielding 11 RCTs involving more than 700 participants (Figure 1) [27-37].

The sample sizes ranged from 28 to 140, including healthy adults, rheumatoid arthritis (RA) patients, breast cancer survivors, type 2 diabetes (T2D) patients, obese adults, older women at risk for Alzheimer’s disease, patients with hypertension/diabetes, and women with psychological distress. The interventions lasted from 6 to 12 weeks. Table 1 summarises the characteristics of the included studies.

**Table 1:** Characteristics of included studies on yoga and gene expression AMPK: Adenosine Monophosphate-Activated Protein Kinase; Bax: Bcl-2-Associated X Protein; Bcl2: B-Cell Lymphoma 2; BEM: Brain Education-Based Meditation; CTLA4: Cytotoxic T-Lymphocyte-Associated Protein 4; CVRFs: Cerebrovascular Risk Factors; DAS28-ESR: Disease Activity Score 28-Erythrocyte Sedimentation Rate; FOXO3: Forkhead Box O3; FoxP3: Forkhead Box P3; IL-1B (or IL1B): Interleukin-1 Beta; IL-6: Interleukin-6; IL-10: Interleukin-10; IL-17: Interleukin-17; INF-gamma: Interferon-Gamma; KLOTHO: Klotho - A gene and protein linked to aging and cellular protection; LDL: Low-Density Lipoprotein; miR-133B: MicroRNA-133B; MME: Membrane Metallo-Endopeptidase; NF-kB (or NFKB2): Nuclear Factor Kappa B; OGG1: 8-Oxoguanine DNA Glycosylase; p53: Tumor Protein p53; PMPS: Post-Mastectomy Pain Syndrome; QoL: Quality of Life; RCT: Randomised Controlled Trial; RA: Rheumatoid Arthritis; RELA: v-Rel Avian Reticuloendotheliosis Viral Oncogene Homolog A; RORyt (or RORγt): RAR-Related Orphan Receptor Gamma t; SCD: Subjective Cognitive Decline; sHLA-G: Soluble Human Leukocyte Antigen-G; SIRT-1: Sirtuin 1; T2D: Type 2 Diabetes; TERT: Telomerase Reverse Transcriptase; TFAM: Transcription Factor A, Mitochondrial; TGF-β: Transforming Growth Factor-Beta; Th17/Treg: T-Helper 17/Regulatory T Cells; TIMP-1: Tissue Inhibitor of Metalloproteinases 1; TNF-α: Tumor Necrosis Factor-Alpha; WHOQOL-BREF: World Health Organization Quality of Life-Brief; YBLI: Yoga-Based Lifestyle Intervention; QoL: Quality of Life

Study	Participants	Yoga Intervention	Gene Expression Outcomes	Additional Outcomes	Limitations
Harkess et al. [27]	28 women with psychological distress	8-week yoga ("moving mindfulness")	Reduced TNF methylation; trend toward increased IL-6 protein	No significant changes in psychological distress	Small sample size; detection issues with protein assays; retrospective methylation analysis
Gautam et al. [28]	140 RA patients	8-week YBLI (asanas, pranayama, meditation)	Increased sHLA-G levels, particularly in low-producing genotypes	Reduced disease activity across genotypes	Small sample size; no active control; lack of participant blinding
Epel et al. [29]	94 healthy women	6-day retreat with yoga, meditation, and self-reflection	Downregulation of stress-related genes (e.g., FOXO3, MME); increased telomerase activity (trend)	Improved subjective well-being, vitality, distress reduction	Combined intervention; short duration; small sample size
Gautam et al. [30]	66 RA patients	8-week YBLI (asanas, pranayama, meditation)	Downregulation of IL-6, TNF-α, CTLA4; upregulation of TGF-β	Reduced disease activity (DAS28-ESR), improved QoL (WHOQOL-BREF)	Lack of active control; small sample size; no long-term follow-up
Gautam et al. [31]	70 RA patients	8-week YBLI (asanas, pranayama, meditation)	Upregulation of AMPK, TIMP-1, KLOTHO, SIRT-1, TFAM	Improved mitochondrial function, reduced oxidative stress, lower DAS28-ESR	Small sample size; lack of participant blinding; intervention intensity
Gautam et al. [32]	64 RA patients	8-week YBLI (asanas, pranayama, meditation)	Downregulation of RORyt, IL-17, IL-6; upregulation of FoxP3, TGF-β	Improved Th17/Treg balance, reduced T cell aging, lower DAS28-ESR	Small sample size; lack of participant blinding; short duration
Saxena et al. [33]	40 breast cancer surgery patients	Anulom-Vilom breathing (90 days)	Upregulation of miR-133B	Reduced PMPS incidence, improved QoL and functional status	Small sample size; single-center data; lack of continuous supervision
Nair et al. [34]	61 T2D patients (45 completed)	10-week yoga (asanas, pranayama)	Upregulation of OGG1	Reduced DNA damage, oxidative stress, improved glycemic control	Small completion size; dropouts; short duration
Sharma et al. [35]	72 obese adults	12-week YBLI (asanas, pranayama, meditation)	Transient increase in TERT at 2 weeks; inconsistent TNF-α changes	No significant changes in anthropometric or physiological parameters	Small sample size; short duration; lack of genome-wide analysis
Grzenda et al. [36]	79 older women with SCD and CVRFs	12-week Kundalini yoga	Altered aging-related gene expression (e.g., INF-gamma, IL-10); prevented eotaxin-1 increase	Improved subjective memory, hippocampal volume, functional connectivity	Small sample size; homogeneous population; short duration
Lee et al. [37]	48 patients with hypertension/diabetes	8-week BEM (meditation-based)	Downregulation of NFKB2, RELA, IL1B	Reduced LDL cholesterol, improved self-reported mental health	Small completion size; high dropout rate; non-blinded design

Yoga Interventions

Interventions included Kundalini yoga [36], yoga-based lifestyle interventions (YBLIs) [28,30-32,35], brain education-based meditation (BEM) [37], Anulom-Vilom breathing [33], and general yoga combining asanas, pranayama, and meditation [27,29,34], with frequencies ranging from daily to twice weekly. Controls ranged from waitlists to active interventions (e.g., exercise and memory training). The variability in yoga styles reflects diverse practices, complicating direct comparisons.

Molecular Outcomes

Five studies reported downregulation of pro-inflammatory genes (e.g., interleukin-6 (IL-6), tumour necrosis factor-alpha (TNF-α), and nuclear factor kappa B (NF-κB)) in RA, breast cancer, and hypertensive populations [27,30-32,37], while four noted upregulation of anti-inflammatory genes (e.g., transforming growth factor-beta (TGF-β), forkhead box P3 (FoxP3), and soluble human leukocyte antigen G (sHLA-G)) [28,30,32,36]. One study showed increased 8-oxoguanine DNA glycosylase (OGG1) expression in T2D, supporting DNA repair relevant to stress-related oxidative damage [34]. Three studies identified aging-related gene changes (e.g., telomerase reverse transcriptase (TERT), KLOTHO, and sirtuin-1 (SIRT-1)), suggesting cellular longevity benefits [31,35,36]. Two studies reported epigenetic effects linked to stress resilience: reduced TNF methylation and increased microRNA-133B (miR-133B) [27,33]. These epigenetic changes may reduce chronic disease risk by modulating inflammatory pathways and enhance stress resilience by stabilising cellular responses to stressors.

Clinical Correlates

Yoga reduced disease activity in RA [28,30-32], improved glycemic control in T2D [34], decreased pain in breast cancer survivors [33], and enhanced subjective memory in at-risk women [36]. Anxiety and distress reduction [29,37], along with physiological improvements in low-density lipoprotein (LDL) cholesterol and antioxidant capacity [36,37], were also observed. Molecular changes (e.g., upregulation of TGF-β) correlate with these outcomes.

Quality Assessment

**Figure 2:**
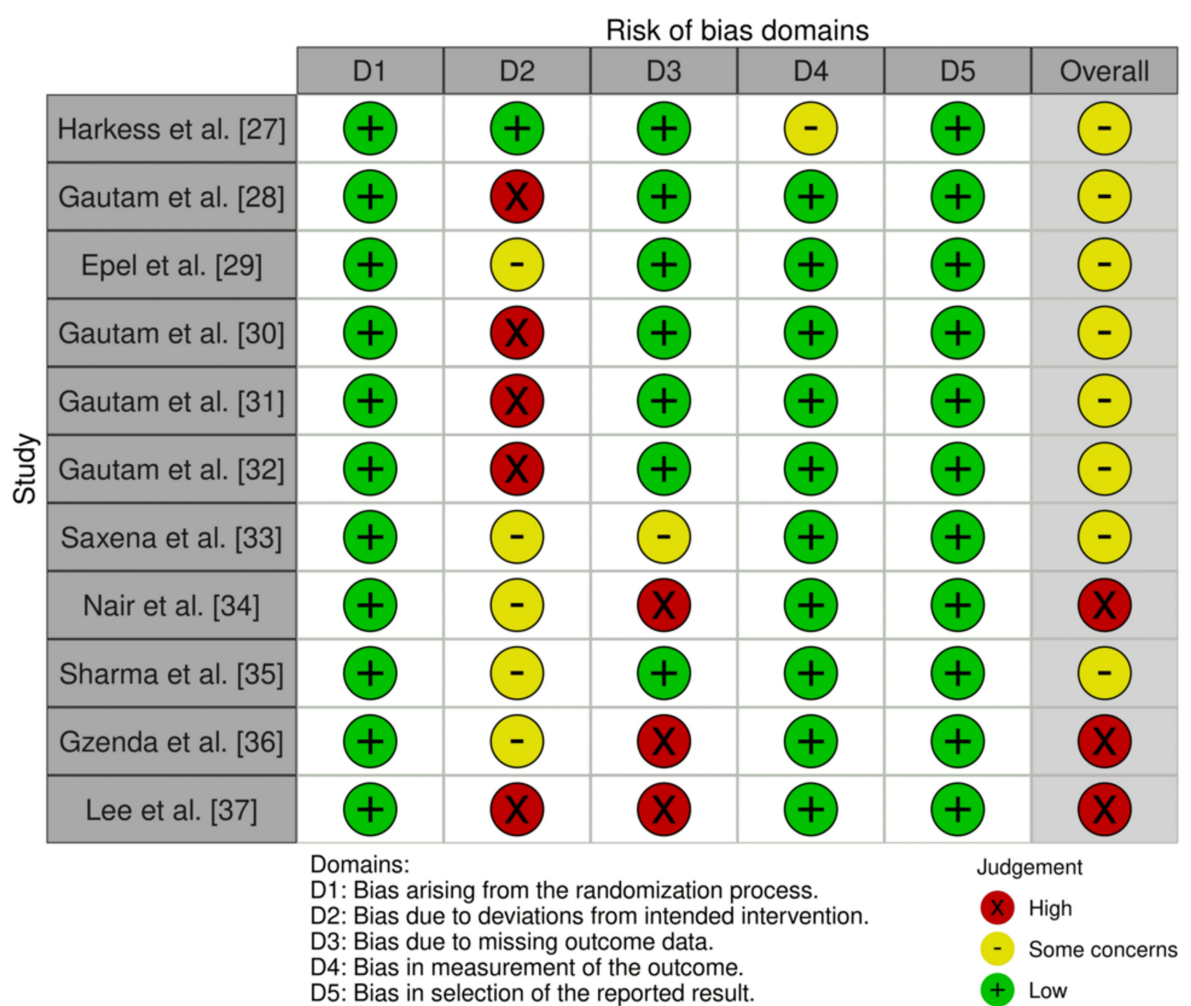
Risk of bias chart

All studies employed randomised designs and utilised validated molecular techniques, such as real-time quantitative polymerase chain reaction (RT-qPCR) and RNA sequencing, to support robust outcome measurements. However, limitations were noted: five studies exhibited a high risk of bias, primarily due to the absence of participant blinding, which is challenging in yoga interventions, while four studies raised concerns related to blinding or incomplete reporting of missing data. Small sample sizes and short intervention durations further constrained statistical power and long-term insights. Despite these risks, the objectivity of molecular outcomes, measured using standardised techniques, mitigates potential biases and enhances the reliability of the findings. Variable control conditions (e.g., waitlist vs. active controls) limited direct comparisons but were addressed through narrative synthesis (Figure 2).

Discussion

This systematic review of 11 RCTs demonstrated the significant influence of yoga on gene expression, particularly genes associated with inflammation, immune function, oxidative stress, and aging, across populations such as RA, T2D, and breast cancer patients. Yoga consistently downregulated pro-inflammatory genes (IL-6, TNF-α, and NF-κB) [27,30-32,37] and upregulated anti-inflammatory genes (TGF-β and FoxP3) [28,30,32,36], suggesting an immune balance. Compared to Buric et al., who noted NF-κB downregulation across mind-body interventions, our yoga-specific focus revealed additional effects on sHLA-G and mitochondrial genes (adenosine monophosphate-activated protein kinase (AMPK) and SIRT-1), broadening molecular insights [24]. 

In RA, yoga reduced IL-17 and RORyt (RAR-related orphan receptor gamma t) levels while increasing sHLA-G levels in low-producing genotypes, correlating with lower disease activity. In T2D, OGG1 upregulation reduces oxidative damage and supports metabolic health [34]. Epigenetic changes (e.g., TNF methylation and miR-133B) indicate the role of yoga in stress resilience, potentially preventing chronic disease [27,33]. 

The clinical advantages of yoga, including reduced RA activity, enhanced glycemic control, and improved quality of life, highlight its therapeutic potential. The YBLI may more effectively target inflammation compared to other modalities, thereby justifying further investigation. The consistency of findings across various studies supports the efficacy of yoga, despite potential biases.

Limitations and future directions

Despite these promising results, several limitations warrant consideration. Many studies had small sample sizes, limiting the generalisability of the findings. The lack of participant blinding in some trials introduces potential bias, particularly for subjective outcomes. Future research should focus on larger, well-powered RCTs with standardised yoga protocols (e.g., a minimum 12-week duration), multi-omics approaches, and diverse populations to confirm sustainability, as well as extended follow-up periods to evaluate the sustainability of gene expression changes. The limited number of epigenetic studies highlights a key research gap, warranting further exploration of yoga’s epigenetic effects. Exploring the effects of yoga on a broader range of genes and pathways, such as those related to neuroplasticity and stress resilience, could clarify the underlying mechanisms. 

## Conclusions

This systematic review demonstrates that yoga interventions modulate gene expression by downregulating pro-inflammatory genes, such as IL-6, TNF-α, and NF-κB, while upregulating anti-inflammatory and immune-regulatory genes, including TGF-β and FoxP3. Additionally, improvements in DNA repair mechanisms (OGG1), mitochondrial function (AMPK and SIRT-1), and epigenetic modifications (TNF methylation and miR-133B) suggest potential benefits for cellular health. These genetic and cellular changes are associated with reduced disease activity, enhanced metabolic outcomes, and an improved quality of life in individuals with chronic conditions. However, the findings are limited by small sample sizes and the short duration of the studies. Nonetheless, yoga shows promise as an adjunct therapeutic approach. Further research, particularly larger and longer-term RCTs, is necessary to elucidate the underlying mechanisms and therapeutic potential of yoga interventions.
